# Supplementary material for: Trajectories of freshwater microbial genomics and greenhouse gas saturation upon glacial retreat
Source: Nat Commun. 2023 Jun 3;14:3234. doi: 10.1038/s41467-023-38806-w (PMC10239486; doi:10.1038/s41467-023-38806-w)

Title: Trajectories of freshwater microbial genomics and greenhouse gas saturation upon glacial retreat

Author list: Jing Wei[1], Laurent Fontaine[1], Nicolas Valiente[1,2], Peter Dörsch[3], Dag O. Hessen[1], Alexander Eiler[1]*

Affiliations:

[1]Department of Biosciences and Centre for Biogeochemistry in the Anthropocene, University of Oslo, 0316 Oslo, Norway

[2]Division of Terrestrial Ecosystem Research, Center of Microbiology and Environmental Systems Science, University of Vienna, 1030 Vienna, Austria

[3]Faculty of Environmental Sciences and Natural Resource Management, Norwegian University of Life Sciences, 1432 Ås, Norway

* Corresponding author: Alexander Eiler, alexander.eiler@ibv.uio.no

Supplementary information

Supplementary tables *3*

Supplementary Table 1. Track table for 16S rRNA bacterial gene amplicons. **3**

Supplementary Table 2. Track table for 18S rRNA eukaryotic gene amplicons. **4**

Supplementary Table 3. Prevalence of significant co-occurrences. **5**

Supplementary Table 4. Statistics on individual assemblies. **5**

Supplementary Table 5. Statistics on contigs from the individual assemblies **6**

Supplementary Table 6. Statistics on co-assembly. **7**

Supplementary Table 7. Statistics on contigs from the co-assembly. **8**

Supplementary Table 8. Overall statistics on bins from the co-assembly. **8**

Supplementary Table 9. Statistics on individual bins from the co-assembly. **9**

Supplementary Table 10. Environmental metadata from the 31 lake systems **10**

Supplementary figures *11*

Supplementary Figure 1. Map of Norway and Svalbard. **11**

Supplementary Figure 2. Generalized additive model (GAM) partial effect splines. **12**

Supplementary Figure 3. Non-parametric multidimensional scaling (NMDS) plots. **12**

Supplementary Figure 4. Heatmaps of bacterial and eukaryotic amplicon sequence variants (ASVs). **13**

Supplementary Figure 5. Results from a general linear latent model (gllvm) on eukaryotic amplicon sequence variants (ASVs). **14**

Supplementary Figure 6. Results from a general linear latent model (gllvm) on bacterial amplicon sequence variants (ASVs). **15**

Supplementary Figure 7. Results from a general linear latent model (gllvm) on selected KEGG families. **16**

Supplementary Figure 8. Results from a general linear latent model (gllvm) on selected traits. **17**

Supplementary Figure 9. Proportion of potential methanotrophic bacterial genera. **18**

Supplementary Figure 10. Example for gating strategy. **19**

Supplementary tables

Supplementary Table 1. Track table for 16S rRNA bacterial gene amplicons.
"Final" represents the number of reads per sample after removal of chimeras (non-chim) and taxonomic groups (non-bacterial, chloroplasts and mitochondrial reads) which were used for downstream analyses.

sample_id	input	filtered	denoisedF	denoisedR	merged	non-chim	final
**FI001**	154249	55850	54580	54639	54310	54057	34227
**FI003**	98353	36053	33164	33395	32366	32366	27033
**FI007**	109868	46366	43697	43802	42986	42975	15262
**FI011**	115356	46161	42201	42411	41180	41178	32109
**FI016**	87327	34701	32981	33147	32643	32634	25717
**SV001**	195573	72027	70501	70659	70033	69075	59961
**SV002**	155539	54555	53880	53909	53764	53691	40475
**SV003**	236801	84899	83062	83331	82338	77472	71224
**SV004**	120930	45290	44981	45000	44939	44900	26033
**SV005**	162410	61032	59072	59146	58544	58192	44770
**SV006**	118482	46523	45154	45243	44956	44940	29625
**SV007**	164783	69106	67719	67844	67409	66803	50162
**SV008**	170598	66039	63936	64175	63233	61067	35655
**SV010**	138077	55618	53387	53531	52897	52382	36896
**SV011**	13678	3715	3434	3427	3361	3361	2912
**SV012**	124804	41187	39504	39621	39065	38104	34337
**SV013**	135936	54141	51076	51260	50075	49481	23107
**SV014**	100253	40664	39612	39626	39318	39316	22662
**SV015**	108453	46315	45769	45836	45715	45574	12045
**SV016**	80471	32452	31528	31619	31425	31425	17881
**SV017**	74830	31633	30825	30879	30701	30605	9229
**SV018**	100263	38883	38531	38536	38470	38420	17145
**SV020**	102308	34041	32663	32761	32343	32338	26722
**SV021**	2729	753	685	685	683	683	443
**SV022**	122312	44796	44082	44090	43964	43887	34697
**SV025**	45929	17488	16893	16944	16785	16755	10322
**SV026**	79558	29043	27652	27703	27370	27370	22512
**SV027**	109650	43471	42957	42984	42834	42834	28745
**SV028**	97969	37947	37243	37303	37040	37027	26658
**SV029**	78873	29432	28567	28590	28405	28405	14923
**SV030**	109542	40425	40117	40119	40047	40044	13751

Supplementary Table 2. Track table for 18S rRNA eukaryotic gene amplicons. ”Final” represents the number of reads per sample after removal of chimeras (non-chim) and taxonomic groups (non-eukaryotic reads) which were used for downstream analyses.

sample_id	input	filtered	denoisedF	denoisedR	merged	nonchim	final
**FI001**	66174	28984	28611	28709	27350	27065	27065
**FI003**	86198	31361	30679	30997	29102	29078	29078
**FI007**	57704	24910	24534	24713	24027	24014	24014
**FI011**	119484	54035	53022	53580	51423	51352	51352
**FI016**	47780	22379	22047	22279	21557	21348	21348
**SV001**	160205	62193	61996	61997	61486	55192	55192
**SV002**	95154	39472	39189	39368	38533	38247	38247
**SV003**	181764	69830	69584	69597	68406	59117	59117
**SV004**	92115	41617	41505	41565	41051	40877	40877
**SV005**	99523	45544	45077	45215	42929	40909	40909
**SV006**	206679	100801	100459	100577	99648	96748	96748
**SV007**	146541	75362	75216	75259	74836	73679	73679
**SV008**	133478	58688	58177	58401	56977	51257	51257
**SV010**	104054	49764	49338	49463	48333	47854	47854
**SV011**	8513	2543	2448	2497	2280	2262	2262
**SV012**	88157	34856	34396	34576	33106	32903	32903
**SV013**	200353	88301	87914	88091	84393	76425	76425
**SV014**	80231	36100	35779	35880	34492	33145	33145
**SV015**	169445	81586	81337	81471	80084	76194	76194
**SV016**	166576	78796	78473	78585	77417	75676	75676
**SV017**	70532	33898	33736	33804	33414	32970	32970
**SV018**	51731	25060	24868	24966	23889	22156	22156
**SV020**	60053	22964	22737	22837	22398	22346	22346
**SV021**	1730	596	563	575	533	533	533
**SV022**	111753	49657	49286	49193	47348	44903	44903
**SV025**	82202	36077	35820	35862	35079	34840	34840
**SV026**	122414	54945	54550	54644	53222	53010	53010
**SV027**	176407	81558	81163	81255	79580	78580	78580
**SV028**	87619	39919	39527	39659	37898	36243	36243
**SV029**	61952	28247	27947	28068	27152	26747	26747
**SV030**	109331	50902	50663	50812	49698	48374	48374

Supplementary Table 3. Prevalence of significant co-occurrences.
Prevalence (percentage %) of significant co-occurrences between and within kingdoms (Bacteria and Eukaryota) as determined by maximal information-based nonparametric exploration[54] revealing similarities in inter- and intra-kingdom co-occurrences.

kingdom	Percentage of sig. co-occurences
**Bacteria**	2.3
**Eukaryota**	3.2
**Between**	2.0

Supplementary Table 4. Statistics on individual assemblies.
These represents the statistics of the assemblies on individual samples used for gene centric analyses.

sample_id	reads	bases	Contigs	ORFs	rRNAs	tRNAs/tmRNAs	orphans	KEGGs
**FI001**	76352015	11298812868	2150046	2667375	3419	12042	1598391	470785
**FI003**	204872670	30413584587	950412	1221795	5006	6081	678255	272330
**FI007**	193948616	28623298602	1570282	2148906	6489	11054	933443	464551
**FI011**	209291026	31127806831	1219269	1638738	7393	7464	797964	384800
**SV001**	191597030	28338597082	1712073	2123421	1755	9017	1405891	326300
**SV002**	206818460	30648775353	1471880	2139766	1844	8645	1511305	296765
**SV003**	167274998	24818473672	1101066	1575809	1854	7325	801573	333780
**SV004**	221887646	32995386440	821926	1034302	717	2516	841694	78071
**SV005**	286414908	42287187965	2901240	3210355	2832	8820	2361282	380079
**SV006**	297078804	43877484261	1426617	1948665	2235	6884	1232195	345242
**SV007**	226988110	33680205801	1330959	1809207	1843	7113	992115	348205
**SV008**	233337150	34675672954	1919810	2620613	3355	12854	1442064	487942
**SV010**	183932292	27306805471	1472374	1921297	3280	10033	1080928	394378
**SV012**	193326296	28841692630	1968537	2770905	4918	15158	1115526	700978
**SV013**	166131216	24397957305	2625123	2995121	4454	8934	2366422	275221
**SV014**	196016900	28928952640	2294479	2999685	2274	7958	2389962	256664
**SV015**	212528672	31376275079	842784	1273957	1153	3476	1084644	86697
**SV016**	168260984	24770973549	1715745	2113586	1991	7263	1622871	206969
**SV017**	229461798	33811886830	1324686	1680727	2240	5313	1142251	227367
**SV018**	172753032	25116269196	1944003	2262620	1193	6093	1969440	109555
**SV020**	214114304	31610359828	2379191	3218094	3573	13234	1580612	667458
**SV021**	259821972	38193426132	2513955	3169841	2259	9460	2414577	284448
**SV022**	188424994	27639514547	2346491	2653170	1605	6833	2094365	262708
**SV025**	172644254	25428742084	2187150	2723259	2013	8515	2078897	257005
**SV026**	189758494	28171842702	2558999	3423245	3887	12849	1548341	796246
**SV027**	242767068	35724616333	2975974	3480374	2291	10612	2757420	274442
**SV028**	190761616	28095270082	2336941	2746123	1924	8898	1932258	359992
**SV029**	274370272	40587111587	2650686	3406422	2684	11177	2614564	339451
**SV030**	158854134	23438173216	1647300	2076711	1438	7721	1452798	230976

Supplementary Table 5. Statistics on contigs from the individual assemblies.

sample_id	contigs	Total length	longest contig	N50	N90	Congruent	Disparity >0	Disparity >= 0.25
**FI001**	2150046	1679210594	557644	950	347	2147952 (99.9%)	2095 (0.1%)	1441 (0.1%)
**FI003**	950412	627719699	445152	706	327	949796 (99.9%)	617 (0.1%)	340 (0.0%)
**FI007**	1570282	1009805849	494339	664	332	1569539 (100.0%)	744 (0.0%)	593 (0.0%)
**FI011**	1219269	859448132	613874	782	329	1217906 (99.9%)	1364 (0.1%)	965 (0.1%)
**SV001**	1712073	1366111996	771697	942	361	1711245 (100.0%)	829 (0.0%)	545 (0.0%)
**SV002**	1471880	1312122718	933739	1237	361	1469967 (99.9%)	1914 (0.1%)	1057 (0.1%)
**SV003**	1101066	906601732	1653268	1001	359	1100082 (99.9%)	985 (0.1%)	639 (0.1%)
**SV004**	821926	560466971	716751	763	322	821512 (99.9%)	415 (0.1%)	134 (0.0%)
**SV005**	2901240	1942386688	1238945	750	327	2900176 (100.0%)	1065 (0.0%)	565 (0.0%)
**SV006**	1426617	1065500501	590453	995	316	1425221 (99.9%)	1397 (0.1%)	876 (0.1%)
**SV007**	1330959	960549695	785307	889	326	1329922 (99.9%)	1038 (0.1%)	629 (0.0%)
**SV008**	1919810	1427880997	701864	864	340	1918604 (99.9%)	1207 (0.1%)	834 (0.0%)
**SV010**	1472374	1070903407	596689	893	328	1471519 (99.9%)	856 (0.1%)	527 (0.0%)
**SV012**	1968537	1473576905	584294	878	347	1966789 (99.9%)	1749 (0.1%)	1345 (0.1%)
**SV013**	2625123	1411308551	454483	548	323	2624706 (100.0%)	418 (0.0%)	291 (0.0%)
**SV014**	2294479	1918843493	1026994	1091	361	2293289 (99.9%)	1191 (0.1%)	645 (0.0%)
**SV015**	842784	784787426	1084123	1860	339	842302 (99.9%)	483 (0.1%)	225 (0.0%)
**SV016**	1715745	1302666623	636583	901	347	1714544 (99.9%)	1202 (0.1%)	746 (0.0%)
**SV017**	1324686	899241632	677971	726	341	1323934 (99.9%)	753 (0.1%)	482 (0.0%)
**SV018**	1944003	1244267800	460295	682	324	1942790 (99.9%)	1214 (0.1%)	707 (0.0%)
**SV020**	2379191	1812753044	469761	888	352	2376829 (99.9%)	2363 (0.1%)	1622 (0.1%)
**SV021**	2513955	2003512665	1280353	1008	353	2512252 (99.9%)	1704 (0.1%)	1094 (0.0%)
**SV022**	2346491	1610239791	581059	788	323	2345235 (99.9%)	1257 (0.1%)	769 (0.0%)
**SV025**	2187150	1641257977	304361	902	345	2185852 (99.9%)	1299 (0.1%)	742 (0.0%)
**SV026**	2558999	1771709787	587263	750	341	2556833 (99.9%)	2167 (0.1%)	1667 (0.1%)
**SV027**	2975974	2103280956	520579	818	333	2974548 (100.0%)	1427 (0.0%)	894 (0.0%)
**SV028**	2336941	1637479561	871054	786	335	2335128 (99.9%)	1814 (0.1%)	1255 (0.1%)
**SV029**	2650686	2049311732	1026964	906	351	2649150 (99.9%)	1537 (0.1%)	988 (0.0%)
**SV030**	1647300	1231651914	481381	893	335	1645662 (99.9%)	1639 (0.1%)	1012 (0.1%)

Supplementary Table 6. Statistics on co-assembly.
These represents the reads and bases used for co-assembly as well as number of open reading frames (ORFs), rRNAs, tRNAs/tmRNAs, orphans and KEGG annotations (KEGGs) obtained from the co-assembled contigs.

sample_id	reads	bases	ORFs	rRNAs	tRNAs/tmRNAs	orphans	KEGGs
**FI001**	171515890	25386092937	5139299	5718	27440	2799429	1080167
**FI003**	204872670	30413584587	3468305	6432	33579	1174768	1267392
**FI007**	193948616	28623298602	3597916	5874	33163	929060	1347327
**FI011**	209291026	31127806831	5048504	6790	41519	1709515	1634123
**SV001**	191597030	28338597082	4018011	5415	25919	1589064	1133052
**SV002**	206818460	30648775353	4098826	5128	19485	2115554	1023364
**SV003**	167274998	24818473672	3997421	5179	23775	1268957	1298263
**SV004**	221887646	32995386440	1139820	4079	8897	497552	384792
**SV005**	286414908	42287187965	7556814	6698	33293	4279689	1485232
**SV006**	297078804	43877484261	4916916	6374	28307	2062780	1315082
**SV007**	226988110	33680205801	4166874	5501	25508	1433787	1265565
**SV008**	233337150	34675672954	5819780	6096	35850	2500024	1468464
**SV010**	183932292	27306805471	5803875	6590	36799	2531547	1464666
**SV012**	193326296	28841692630	4698437	6167	37765	1350918	1507127
**SV013**	166131216	24397957305	3777788	6439	28011	1406026	1176559
**SV014**	196016900	28928952640	6200855	5877	24458	3771317	1105635
**SV015**	212528672	31376275079	2839832	4988	12131	1754121	525027
**SV016**	168260984	24770973549	5386679	5718	22821	3189874	1008250
**SV017**	229461798	33811886830	3518842	5881	21298	1562766	970504
**SV018**	172753032	25116269196	3776197	4392	11587	2667429	493312
**SV020**	214114304	31610359828	5046158	5737	31285	1852852	1473853
**SV021**	259821972	38193426132	6702346	6262	27615	4044010	1190309
**SV022**	188424994	27639514547	5464814	5202	18208	3533572	897279
**SV025**	172644254	25428742084	5146048	5408	20096	3155457	901790
**SV026**	189758494	28171842702	5026961	6310	32852	1808721	1441068
**SV027**	242767068	35724616333	5999722	5560	21173	3925227	927698
**SV028**	190761616	28095270082	6372159	5687	23330	4000314	1049993
**SV029**	274370272	40587111587	7185810	6396	32234	4349354	1264616
**SV030**	158854134	23438173216	4568878	4395	15860	2880101	729123
**Assembly**	6024953606	890312435696	536734552	253721	2558264	324925193	81319509

Supplementary Table 7. Statistics on contigs from the co-assembly.

	Coassembly
**Number of contigs**	6704263
**Total length**	15900628689
**Longest contig**	676008
**Shortest contig**	1000
**N50**	2635
**N90**	1161
**Contigs at superkingdom (k) rank**	2997852 (44.7%) in 4 superkingdoms
**Contigs at phylum (p) rank**	2611829 (39.0%), in 219 phyla
**Contigs at class (c) rank**	2115438 (31.6%), in 280 classes
**Contigs at order (o) rank**	1725599 (25.7%), in 727 orders
**Contigs at family (f) rank**	1382099 (20.6%), in 1565 families
**Contigs at genus (g) rank**	937851 (14.0%), in 4914 genera
**Contigs at species (s) rank**	446916 (6.7%), in 2909 species
**Congruent**	6592740 (98.3%)
**Disparity >0**	111524 (1.7%)
**Disparity >= 0.25**	110136 (1.6%)

Supplementary Table 8. Overall statistics on bins from the co-assembly.

	Coassembly
**Number of bins**	1981
**Complete >= 50%**	200
**Complete >= 75%**	21
**Complete >= 90%**	1
**Contamination < 10%**	1955
**Contamination >= 50%**	2
**Congruent bins**	932
**Disparity >0**	1049
**Disparity >= 0.25**	471
**Hi-qual bins (>90% complete,<10% contam)**	0
**Good-qual bins (>75% complete,<10% contam)**	17

Supplementary Table 9. Statistics on individual bins from the co-assembly.

Bin.ID	Tax	Length	GC.perc	Num.contigs	Disparity	Completeness	Contamination	Strain heterogeneity	Average coverage %
**metabat2.1000**	k_Bacteria;n_Terrabacteria group;p_Actinobacteria;c_Actinobacteria;n_unclassified Actinobacteria (class);s_Actinobacteria bacterium	2821442	63.65	161	0.028	89.61	0.63	50	0.0656
**metabat2.1750**	k_Bacteria;n_Bacteria incertae sedis;n_Bacteria candidate phyla;n_Candidatus Dependentiae;n_unclassified Candidatus Dependentiae;s_Candidatus Dependentiae bacterium	951254	33.48	17	0	88.71	0.34	100	0.0201
**metabat2.762**	k_Bacteria;p_Proteobacteria;c_Gammaproteobacteria;o_Aeromonadales;f_Aeromonadaceae;g_Aeromonas	3324955	60	381	0.005	84.66	1.41	66.67	0.0104
**metabat2.1193**	k_Bacteria;n_PVC group;p_Planctomycetes;c_Planctomycetia;o_Planctomycetales;f_Planctomycetaceae;n_unclassified Planctomycetaceae;s_Planctomycetaceae bacterium	2391958	57.22	142	0.024	83.4	0	0	0.0437
**metabat2.799**	k_Bacteria;n_FCB group;n_Bacteroidetes/Chlorobi group;p_Bacteroidetes;c_Flavobacteriia;o_Flavobacteriales;f_Flavobacteriaceae;g_Flavobacterium	2329431	37.62	225	0.019	83.36	0.42	33.33	0.0114
**metabat2.921**	k_Bacteria;n_FCB group;p_Gemmatimonadetes;c_Gemmatimonadetes;o_Gemmatimonadales;f_Gemmatimonadaceae;g_Gemmatimonas;n_unclassified Gemmatimonas;s_Gemmatimonas sp.	2982943	64.99	265	0.239	82.76	0	0	0.0219
**metabat2.1354**	k_Bacteria;p_Proteobacteria;c_Alphaproteobacteria;o_Sphingomonadales;f_Sphingomonadaceae;g_Sandarakinorhabdus	2488492	64.68	274	0.125	81.25	2.6	75	0.0285
**metabat2.479**	k_Bacteria;n_FCB group;n_Bacteroidetes/Chlorobi group;p_Bacteroidetes;n_unclassified Bacteroidetes;s_Bacteroidetes bacterium	1940449	43.86	199	0.033	79.33	0.68	100	0.0118
**metabat2.1778**	k_Bacteria;n_FCB group;n_Bacteroidetes/Chlorobi group;p_Bacteroidetes;c_Flavobacteriia;o_Flavobacteriales;f_Flavobacteriaceae;g_Flavobacterium;s_Flavobacterium sangjuense	2036895	34.78	133	0.216	79.16	4.61	12.5	0.0343
**metabat2.1954**	k_Bacteria;p_Proteobacteria;c_Alphaproteobacteria;o_Sphingomonadales;f_Sphingomonadaceae;g_Blastomonas	2252242	64.08	376	0.046	78.41	2.01	85.71	0.0277
**metabat2.761**	k_Bacteria;n_FCB group;n_Bacteroidetes/Chlorobi group;p_Bacteroidetes;c_Flavobacteriia;o_Flavobacteriales;f_Flavobacteriaceae;g_Flavobacterium	1779360	32.79	88	0	77.08	0.23	50	0.0577
**metabat2.64**	k_Bacteria;n_Terrabacteria group;n_Cyanobacteria/Melainabacteria group;p_Cyanobacteria;o_Synechococcales;f_Synechococcaceae;g_Synechococcus;n_unclassified Synechococcus;s_Synechococcus sp.	1821421	67.93	202	0.267	76.76	2.59	33.33	0.1074
**metabat2.592**	k_Bacteria;n_Terrabacteria group;p_Actinobacteria;c_Actinobacteria;n_unclassified Actinobacteria (class);s_Actinobacteria bacterium	1020115	55.48	47	0.053	76.45	0	0	0.0118
**metabat2.1867**	k_Bacteria;n_Terrabacteria group;p_Actinobacteria;c_Actinobacteria;o_Corynebacteriales;f_Mycobacteriaceae;g_Mycobacterium	2532316	65.76	340	0.096	75.86	0.63	60	0.0120
**metabat2.1077**	k_Bacteria;n_Terrabacteria group;n_Cyanobacteria/Melainabacteria group;p_Cyanobacteria	1763772	44.7	293	0.007	75.77	1.68	28.57	0.0096
**metabat2.900**	k_Bacteria;n_Terrabacteria group;p_Actinobacteria;c_Actinobacteria;o_Corynebacteriales;f_Mycobacteriaceae;g_Mycobacterium;n_unclassified Mycobacterium;s_Mycobacterium sp.	2143414	66.31	193	0.068	75.67	0.78	62.5	0.0807
**metabat2.816**	k_Bacteria;n_FCB group;n_Bacteroidetes/Chlorobi group;p_Bacteroidetes;c_Cytophagia;o_Cytophagales;f_Cyclobacteriaceae;g_Aquiflexum;n_unclassified Aquiflexum;s_Aquiflexum sp.	3259978	39.71	471	0.09	74.98	1.01	16.67	0.0089
**metabat2.911**	k_Bacteria;p_Proteobacteria;c_Alphaproteobacteria;n_unclassified Alphaproteobacteria;s_Alphaproteobacteria bacterium	1070596	32.7	191	0.14	73.63	2.73	100	0.0157
**metabat2.1276**	k_Bacteria	413541	45.82	31	0	73.56	0	0	0.0171
**metabat2.32**	k_Bacteria;p_Proteobacteria;c_Alphaproteobacteria;o_Rhodobacterales;f_Rhodobacteraceae	2697815	61.96	493	0.087	72.3	3.37	53.85	0.0139
**metabat2.976**	k_Bacteria;n_FCB group;n_Bacteroidetes/Chlorobi group;p_Bacteroidetes;c_Flavobacteriia;o_Flavobacteriales;f_Flavobacteriaceae;g_Flavobacterium	2033799	32.51	245	0.034	72.15	3.39	26.67	0.0253
**metabat2.1685**	k_Bacteria;p_Proteobacteria;c_Betaproteobacteria;o_Burkholderiales;n_Burkholderiales genera incertae sedis;g_Aquabacterium;s_Aquabacterium commune	2065665	65.33	116	0.087	71.69	0	0	0.0243
**metabat2.898**	k_Bacteria;p_Proteobacteria;c_Betaproteobacteria;o_Burkholderiales;f_Comamonadaceae	2190042	64.87	328	0.022	71.49	3.55	38.89	0.0208
**metabat2.1091**	k_Bacteria;n_PVC group;p_Planctomycetes;c_Planctomycetia	2320875	65.43	188	0.094	71.15	0.85	20	0.0201
**metabat2.1811**	k_Bacteria;n_Terrabacteria group;n_Cyanobacteria/Melainabacteria group;p_Cyanobacteria;o_Nostocales;f_Nostocaceae;g_Nostoc	4296276	40.57	729	0.028	70.56	1.36	70	0.0602
**metabat2.558**	k_Bacteria;n_FCB group;n_Bacteroidetes/Chlorobi group;p_Bacteroidetes;c_Chitinophagia;o_Chitinophagales;f_Chitinophagaceae;n_unclassified Chitinophagaceae;s_Chitinophagaceae bacterium	1795331	35.81	41	0.067	70.46	0.75	60	0.0285
**metabat2.467**	k_Bacteria;p_Proteobacteria;c_Betaproteobacteria;o_Burkholderiales;f_Oxalobacteraceae	2127614	55.75	241	0.035	69.23	0.79	66.67	0.0111
**metabat2.889**	k_Bacteria;n_FCB group;n_Bacteroidetes/Chlorobi group;p_Bacteroidetes;c_Sphingobacteriia;n_unclassified Sphingobacteriia;s_Sphingobacteriia bacterium	1504574	30.03	269	0.133	68.74	0.21	100	0.0175
**metabat2.1320**	k_Bacteria;p_Proteobacteria;c_Alphaproteobacteria;o_Rhodobacterales;f_Rhodobacteraceae	2117186	67.35	127	0	68.39	2.15	75	0.0574
**metabat2.407**	k_Bacteria;p_Proteobacteria;c_Alphaproteobacteria;o_Sphingomonadales;f_Sphingomonadaceae	1938659	67.82	354	0.31	68.08	2.9	15	0.0179
**metabat2.1957**	k_Bacteria;n_FCB group;n_Bacteroidetes/Chlorobi group;p_Bacteroidetes;c_Flavobacteriia;o_Flavobacteriales;f_Flavobacteriaceae;g_Flavobacterium	1635588	34.56	242	0	67.69	1.09	28.57	0.0124
**metabat2.1707**	k_Bacteria;p_Proteobacteria;c_Betaproteobacteria;o_Burkholderiales;f_Oxalobacteraceae;n_unclassified Oxalobacteraceae;s_Oxalobacteraceae bacterium	1269423	57.82	133	0.068	67.31	0.75	50	0.0237
**metabat2.1658**	k_Bacteria;p_Proteobacteria;c_Alphaproteobacteria;o_Sphingomonadales;f_Sphingomonadaceae	1867082	64.34	305	0.029	67.05	2.23	76.92	0.0331
**metabat2.1016**	k_Bacteria;p_Proteobacteria;c_Alphaproteobacteria;o_Rhodobacterales;f_Rhodobacteraceae	1876290	56.89	248	0.032	66.97	1.56	100	0.0307
**metabat2.321**	k_Bacteria;n_Terrabacteria group;p_Actinobacteria;c_Actinobacteria	1809716	58.8	288	0.056	66.95	0	0	0.0081
**metabat2.1032**	k_Bacteria;n_PVC group;p_Planctomycetes;c_Planctomycetia	2323546	64.56	175	0.048	66.46	1.17	100	0.0151
**metabat2.86**	k_Bacteria;p_Proteobacteria;c_Alphaproteobacteria;o_Rhodobacterales;f_Hyphomonadaceae;g_Hyphomonas	2301131	64.23	282	0.387	66.38	3.56	8.33	0.0104
**metabat2.400**	k_Bacteria;n_Terrabacteria group;n_Cyanobacteria/Melainabacteria group;p_Cyanobacteria;o_Synechococcales;f_Synechococcaceae;g_Synechococcus	1565718	67.86	251	0.064	66.32	1.45	62.5	0.0124
**metabat2.1151**	k_Bacteria;p_Proteobacteria;c_Gammaproteobacteria;o_Xanthomonadales;f_Xanthomonadaceae;g_Arenimonas	1553427	69.53	246	0.049	66.22	3.24	61.54	0.0497
**metabat2.987**	k_Bacteria;n_PVC group;p_Planctomycetes;n_unclassified Planctomycetes;s_Planctomycetes bacterium	2152462	59.54	128	0.288	65.89	0.39	100	0.1338
**metabat2.668**	k_Bacteria;p_Nitrospirae;n_unclassified Nitrospirae;s_Nitrospirae bacterium	1555391	63.27	244	0.42	65.77	1.77	27.27	0.0126
**metabat2.96**	k_Bacteria	595883	34.54	88	0	65.52	0	0	0.0073
**metabat2.1064**	k_Bacteria;n_Terrabacteria group;n_Cyanobacteria/Melainabacteria group;p_Cyanobacteria;o_Synechococcales;f_Leptolyngbyaceae;g_Phormidesmis	3187499	49.41	292	0.32	65.35	1.31	28.57	0.0084
**metabat2.996**	k_Bacteria;n_Terrabacteria group;n_Cyanobacteria/Melainabacteria group;p_Cyanobacteria;o_Synechococcales;f_Synechococcaceae;g_Synechococcus	1017836	51.42	117	0.03	65.33	0	0	0.0453
**metabat2.1934**	k_Bacteria;p_Proteobacteria;c_Betaproteobacteria;o_Burkholderiales	3654868	67.61	680	0.072	65.25	2.15	22.22	0.0181
**metabat2.34**	k_Bacteria;p_Proteobacteria;c_Betaproteobacteria;o_Burkholderiales;n_unclassified Burkholderiales;s_Burkholderiales bacterium	1676166	68.27	147	0.015	65.23	1.28	20	0.0474
**metabat2.1749**	k_Bacteria;n_FCB group;n_Bacteroidetes/Chlorobi group;p_Bacteroidetes	2322322	36.17	415	0.044	64.96	0.51	0	0.0146
**metabat2.1610**	k_Bacteria;n_Terrabacteria group;p_Chloroflexi;c_Chloroflexia	1873088	53.61	75	0.111	64.21	1.17	50	0.0710
**metabat2.1770**	k_Bacteria;n_PVC group;p_Planctomycetes;n_unclassified Planctomycetes;s_Planctomycetes bacterium	2717651	60.27	409	0	64.2	1.56	0	0.0081

Supplementary Table 10. Environmental metadata from the 31 lake systems. This can also be obtained from the OSF data repository (www.osf.io) under DOI: 10.17605/OSF.IO/PNWKS.

sample_id	sample_name	water_loc_code	sample_date	comments	locality	altitude	north	east	gl_dist	DNA_conc	Ccounts	Temp	Cond	pH	filtered_water	TOC	DOC	TN	DN	TP	DP	O2	N2	CO2	CH4	N2O	bird	N2_sat	O2_sat	CO2_sat	CH4_sat
									[m]	[ng/µl]	[cells/ml]	[C]			[ml]	[mg/	[mg/L	[mg/L]	[mg/L]	[ng/L]	[ng/L]	[µM]	[µM]	[µM]	[nM]	[nM]		[%]	[%]	[%]	[%]
**SV001**	Hotellnesset	HAV-88683	7/8/2019	Daphnia and bird colonies Tarn Goose	Longyearbyn	1	78.2495	15.4914	10000	12.3	232139	12.2	NA	NA	480	2.878	4.032	0.305	0.319	9	7	NA	NA	NA	NA	NA	2	NA	NA	NA	NA
**SV002**	Isdammen	HAV-28272	7/8/2019	Lepidyrus arcticus high turbidity	Longyearbyn	1	78.2157	15.7202	10000	9.36	131857	10.2	NA	NA	240	2.93	3.33	0.022	0.054	3	3	NA	NA	NA	NA	NA	1	NA	NA	NA	NA
**SV003**	West/East	NA	7/8/2019	Daphnia bird colonies	Longyearbyn	1	78.219	15.6961	10000	94	63908	10.9	NA	NA	720	1.831	3.555	0.424	0.337	16	8	NA	NA	NA	NA	NA	3	NA	NA	NA	NA
**SV004**	Gruve7	NA	7/8/2019		Longyearbyn	400	78.1567	16.0327	5000	5.78	102050	9.4	NA	NA	720	0.527	2.412	0.036	0.6462	2	2	NA	NA	NA	NA	NA	0	NA	NA	NA	NA
**SV005**	Tvillingvatten	NA	8/8/2019		Broggerdalen	33	78.9159	11.8755	1300	11.2	140698	9.8	181.6	7.82	960	15.22	3.295	0.046	0.052	2	2	337.95	625.47	766.21	9.36	12.68	1	111.8	108.5	173.4	289.6
**SV006**	Storvatnet	NA	9/8/2019	Daphnia	Broggerdalen	30	78.9239	11.8783	2250	14.7	261441	9	194.3	8.18	360	3.891	10.84	0.3213	0.348	2	2	431.79	905.95	966.40	274.04	13.56	2	159.8	136.5	97.8	8340.9
**SV007**	Solvatnet	HAV-28270	8/8/2019	Daphnia Lepidyrus potential large algae colony	Broggerdalen	2	78.9258	11.9405	3600	11.8	504190	14.8	332	8.25	200	7.739	8.607	0.815	0.884	19	9	302.94	561.95	1148.06	1136.34	13.38	3	108.8	106.6	105.5	38771.4
**SV008**	Brandallaguna	NA	9/8/2019		Broggerdalen	1	78.9442	11.8631	3600	14.1	253105	9.9	299	9.01	480	2.578	4.546	0.201	0.279	9	2	417.94	908.82	998.58	906.99	13.03	3	162.7	134.4	15.0	28108.1
**SV010**	Knudsenheia1	NA	8/8/2019	Daphnia Lepityrus	Broggerdalen	26	78.942	11.821	3100	8.18	455690	8.5	239	8.44	480	5.059	6.482	0.359	0.426	11	0	346.70	637.37	444.54	150.65	13.81	3	111.5	108.6	48.9	4539.4
**SV011**	Lovenbreendalen5	NA	11/8/2019	Glacier front	Lovenbreendalen	46	78.8959	12.064	30	5.96	96998	5.2	16.5	6.78	200	1.591	1.941	0.006	0.01	3	0	501.84	1132.01	137.94	8.14	17.77	0	186.1	147.6	184.9	174.7
**SV012**	Goose pond	NA	8/8/2019	Daphnia	Broggerdalen	3	78.9258	11.9231	3400	8.96	393298	6.2	244	8.71	540	3.964	11.18	0.24	0.317	3	0	370.95	669.41	1382.33	1638.41	17.29	3	112.8	111.2	41.0	47112.6
**SV013**	Permafrostpond	NA	8/8/2019	Thawpond with ice	Broggerdalen	3	78.9261	11.9205	3400	48	241233	2.8	468	7.59	300	2.169	2.217	0.273	0.164	9	2	391.55	715.90	3122.85	82.53	26.50	3	113.9	109.9	1121.0	2211.4
**SV014**	Broggerdalen1	NA	9/8/2019	Daphnia Moses bird-traces farces	Broggerdalen	50	78.9132	11.8403	790	8.2	220520	9.2	208	7.93	960	6.702	1.333	0.048	0.072	0	0	336.39	621.58	1011.87	4.62	13.29	1	110.0	106.8	180.0	141.1
**SV015**	Broggerdalen2	NA	9/8/2019		Broggerdalen	42	78.9127	11.8299	800	8.84	107355	9.2	229	7.77	840	10.51	5	0.04	0.061	0	0	342.40	632.12	1132.53	4.63	16.39	0	111.9	108.7	287.4	141.6
**SV016**	Broggerdalen3	NA	9/8/2019	Crack in filter	Broggerdalen	43	78.9131	11.84	790	6.48	82600	9.9	87.7	7.89	600	7.106	14.09	0.14	0.14	0	0	436.19	941.91	574.81	12.48	13.77	0	168.6	140.3	110.3	386.6
**SV017**	Lovenbreendalen6	NA	11/8/2019		Lovenbreendalen	44	78.8986	12.0628	300	7.72	78559	6.9	216	7.58	1200	1.314	1.049	0.003	0.007	0	0	363.80	669.57	1001.38	4.84	14.60	0	114.0	110.5	434.8	136.4
**SV018**	Lovenbreendalen7	NA	11/8/2019		Lovenbreendalen	38	78.9	12.0666	550	21.4	80074	5.7	263	7.51	1200	5.229	1.235	0.025	0.021	0	0	375.56	689.69	1163.89	4.09	15.79	0	115.1	111.5	580.3	112.4
**SV020**	Gluudneset	NA	11/8/2019	Daphnia	Lovenbreendalen	1	78.9133	12.0628	1860	7.58	158885	9.8	1410	7.83	480	5.326	5.491	0.306	0.377	2	0	336.54	623.09	1252.66	3.82	12.82	3	111.3	108.0	327.6	114.1
**SV021**	Lovenbreendalen1	NA	11/8/2019	Daphnia	Lovenbreendalen	35	78.901	12.0682	670	10.2	134383	9.3	235	7.71	1200	1.216	3.096	0.061	0.06	0	0	424.04	949.33	971.32	5.10	13.17	0	168.2	134.8	326.7	150.9
**SV022**	Lovenbreendalen2	NA	11/8/2019	Daphnia	Lovenbreendalen	24	78.9043	12.0637	970	17.8	116954	8.3	150.4	7.7	900	1.415	3.48	0.087	0.084	0	0	396.76	781.95	468.87	6.54	13.74	0	136.3	123.8	155.5	189.6
**SV025**	Sarsvatnet	NA	10/8/2019	Daphnia	Ossian Sarsfjellet	105	78.9503	12.4984	940	4.82	126553	9.7	175.3	7.72	960	13.23	1.494	0.079	0.094	0	0	339.35	626.09	1005.10	33.93	13.22	0	112.3	109.2	320.4	1111.4
**SV026**	OssianSars1	NA	10/8/2019	Methane bubbles	Ossian Sarsfjellet	120	78.9543	12.4962	1390	9.46	339494	10.4	198.2	8.65	300	8.171	5.858	0.387	0.451	0	0	340.08	622.87	948.17	1942.55	12.25	0	133.6	138.5	37.2	61078.7
**SV027**	OssianSars2	NA	10/8/2019	No birdtraces	Ossian Sarsfjellet	130	78.9583	12.504	1150	9.4	149287	10.3	197.8	8.08	1200	1.695	4.747	0.055	0.072	2	0	334.42	617.89	1370.72	249.10	14.41	0	111.9	108.9	199.4	7959.6
**SV028**	OssianSars3	NA	10/8/2019	No birdtraces	Ossian Sarsfjellet	151	78.9545	12.5331	590	21.4	160654	10.6	164.1	8.05	1200	3.161	2.978	0.056	0.07	0	0	453.39	1018.03	1153.40	129.83	12.10	0	185.3	148.4	179.4	4087.4
**SV029**	OssianSars4	NA	10/8/2019	Daphnia	Ossian Sarsfjellet	150	78.9522	12.5365	510	13.6	109123	10.8	202	7.9	900	7.694	7.098	0.077	0.093	0	0	336.77	614.82	810.25	13.15	12.06	0	112.3	110.6	175.1	415.8
**SV030**	OssianSars5	NA	10/8/2019	Bird farces Daphnia	Ossian Sarsfjellet	133	78.9512	12.5353	450	7.84	125037	10.2	217	7.89	780	1.854	1.726	0.063	0.053	0	0	335.89	621.35	1278.74	8.03	12.59	1	112.4	109.1	283.7	250.9
**FI001**	Finsevatnet East	012-27929	23/08/2019	NA	Finse	1215	60.59322	7.50961	3300	12.8	34101	10.1	5	6.6	720	8.08	6.072	0.062	0.029	10	8	371.72	832.34	64.41	7.05	12.61	0	154.3	123.9	108.9	220.5
**FI003**	FI003	NA	23/08/2019	NA	Finse	1250	60.57889	7.52092	2070	9.2	656760	9.2	1	7.6	840	10.34	4.409	0.152	0.097	9	9	392.51	837.00	103.19	70.03	13.44	0	152.9	128.7	34.6	2151.3
**FI007**	FI007	NA	23/08/2019	NA	Finse	1420	60.56056	7.50808	120	10.9	88031	8.5	106	7.7	720	16.87	1.879	0.031	0.027	8	7	338.06	624.54	396.97	4.07	13.93	0	112.8	109.4	128.3	123.2
**FI011**	FI011	NA	23/08/2019	NA	Finse	1321	60.56133	7.50939	240	13	71991	9.6	56	8	960	0.8	0.761	0.03	0.03	8	6	330.20	611.09	233.46	6.03	13.57	0	112.4	109.0	37.9	186.6
**FI016**	FI016	NA	23/08/2019	NA	Finse	1260	60.56497	7.51156	530	6.8	60624	9	103	6.3	1200	0.69	0.635	0.08	0.071	7	5	345.93	627.82	154.81	6.21	16.98	0	114.3	113.0	451.5	189.9

Supplementary figures

Supplementary Figure 1. Map of Norway and Svalbard. The maps were drawn in ArcGIS 10.7 for Desktop, Esri Inc., http://www.esri.com.

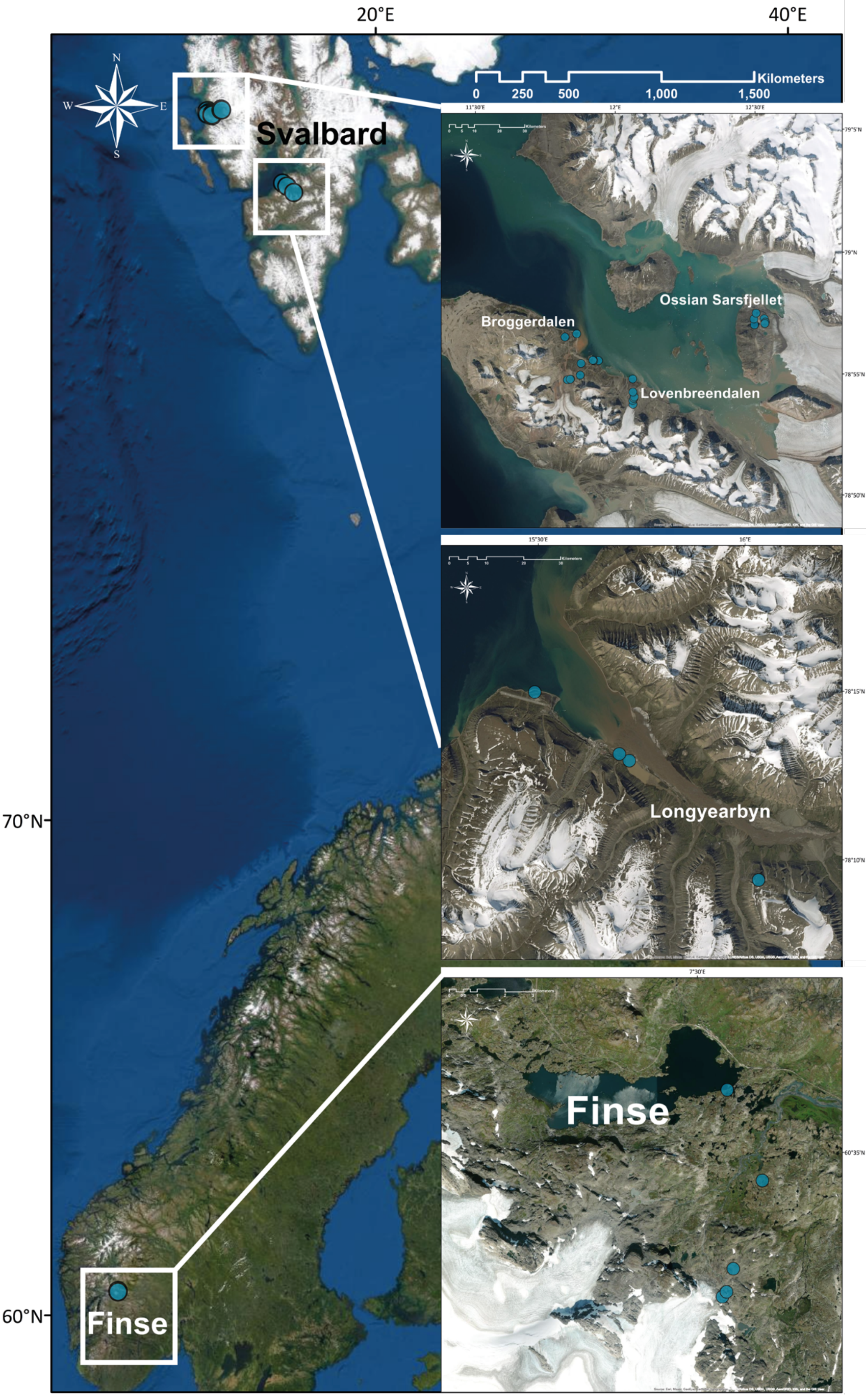

Supplementary Figure 2. Generalized additive model (GAM) partial effect splines. GAM partial effect splines. (as shown by red lines) for significant variables modeling CO2 saturation including dissolved organic carbon (DOC; A) and total nitrogen concentrations (TN, B). Standard errors are indicated by dotted lines while data points are shown by open circles.

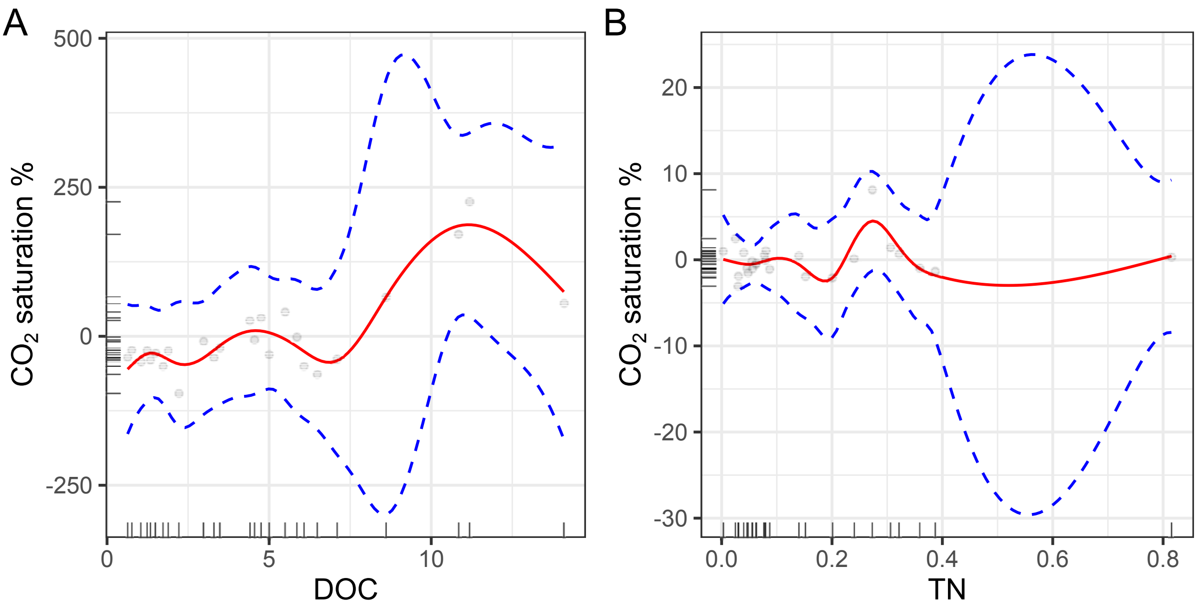

Supplementary Figure 3. Non-parametric multidimensional scaling (NMDS) plots. NMDS plots based on bacterial (A) and eukaryotic (B) amplicon sequence variants (ASV) tables as well as functional profiles as assessed by KEGG annotations (C). Symbols indicates the chronosequence and color distance from glacier. Stress values are given for each plot.

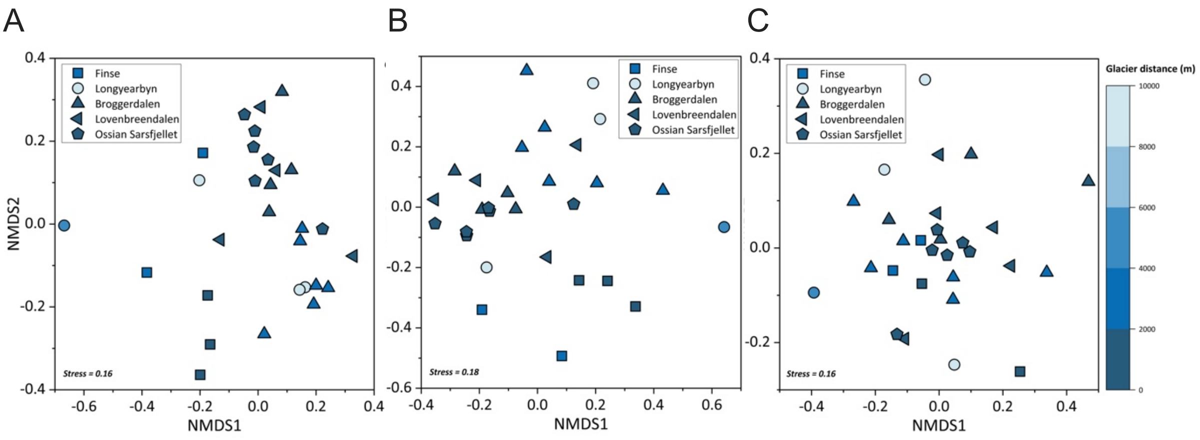

Supplementary Figure 4. Heatmaps of bacterial and eukaryotic amplicon sequence variants (ASVs).
Heatmaps of bacterial (A) and eukaryotic (B) ASV tables.

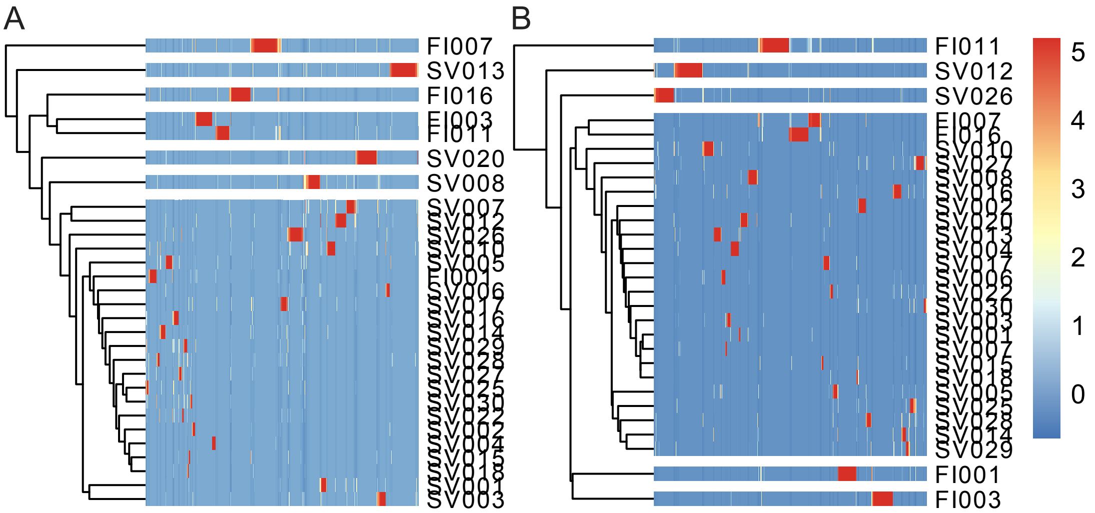

Supplementary Figure 5. Results from a general linear latent model (gllvm) on eukaryotic amplicon sequence variants (ASVs).
Plots of Dunn-Smyth residuals, also known as randomized quantile residuals, are shown, including a plot of residuals against linear predictors of fitted values (A), a Normal Q-Q plot of residuals with a simulated point-wise 95% confidence interval envelope (B), residuals against row index (C) and column index (D) and scale-location plot (E). The estimated coefficients for predictors and their 95% confidence intervals (F – distance from glaciers (gl_dist), G – Dissolved organic carbon (DOC), H – Dissolved nitrogen (DN), I – Total phosphor (TP) and J – bird impact), allow to study the nature of effects of environmental variables on amplicon sequence variants (ASVs). Point estimates (ticks) for coefficients of the environmental variables and their 95% confidence intervals (lines) are given, with those colored in black denoting intervals not containing zero.

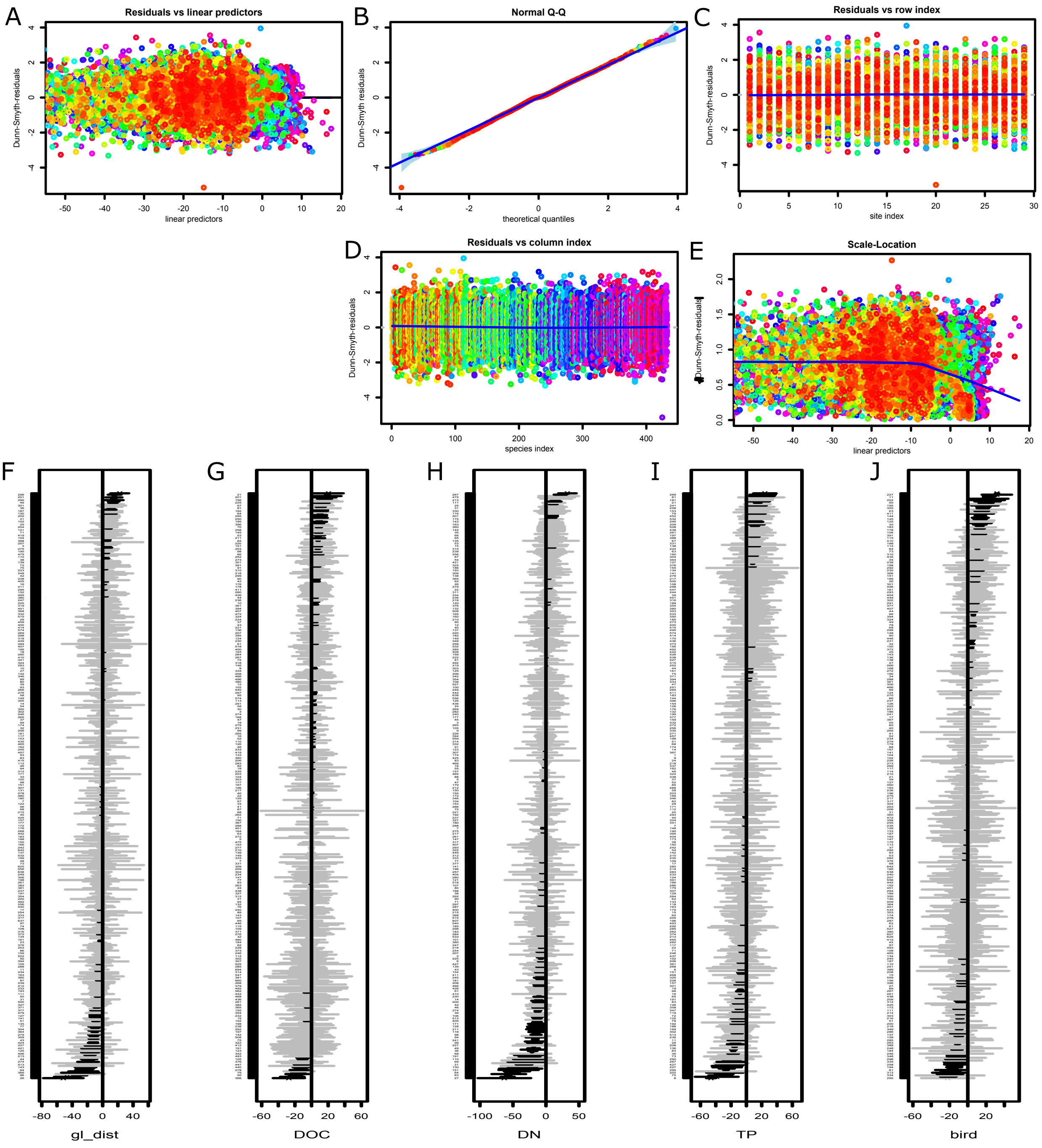

Supplementary Figure 6. Results from a general linear latent model (gllvm) on bacterial amplicon sequence variants (ASVs).
Plots of Dunn-Smyth residuals, also known as randomized quantile residuals, are shown, including a plot of residuals against linear predictors of fitted values (A), a Normal Q-Q plot of residuals with a simulated point-wise 95% confidence interval envelope (B), residuals against row index (C) and column index (D) and scale-location plot (E). The estimated coefficients for predictors and their 95% confidence intervals (F – distance from glaciers (gl_dist), G – Dissolved organic carbon (DOC), H – Dissolved nitrogen (DN), I – Total phosphor (TP) and J – bird impact), allow to study the nature of effects of environmental variables on amplicon sequence variants (ASVs). Point estimates (ticks) for coefficients of the environmental variables and their 95% confidence intervals (lines) are given, with those colored in black denoting intervals not containing zero.

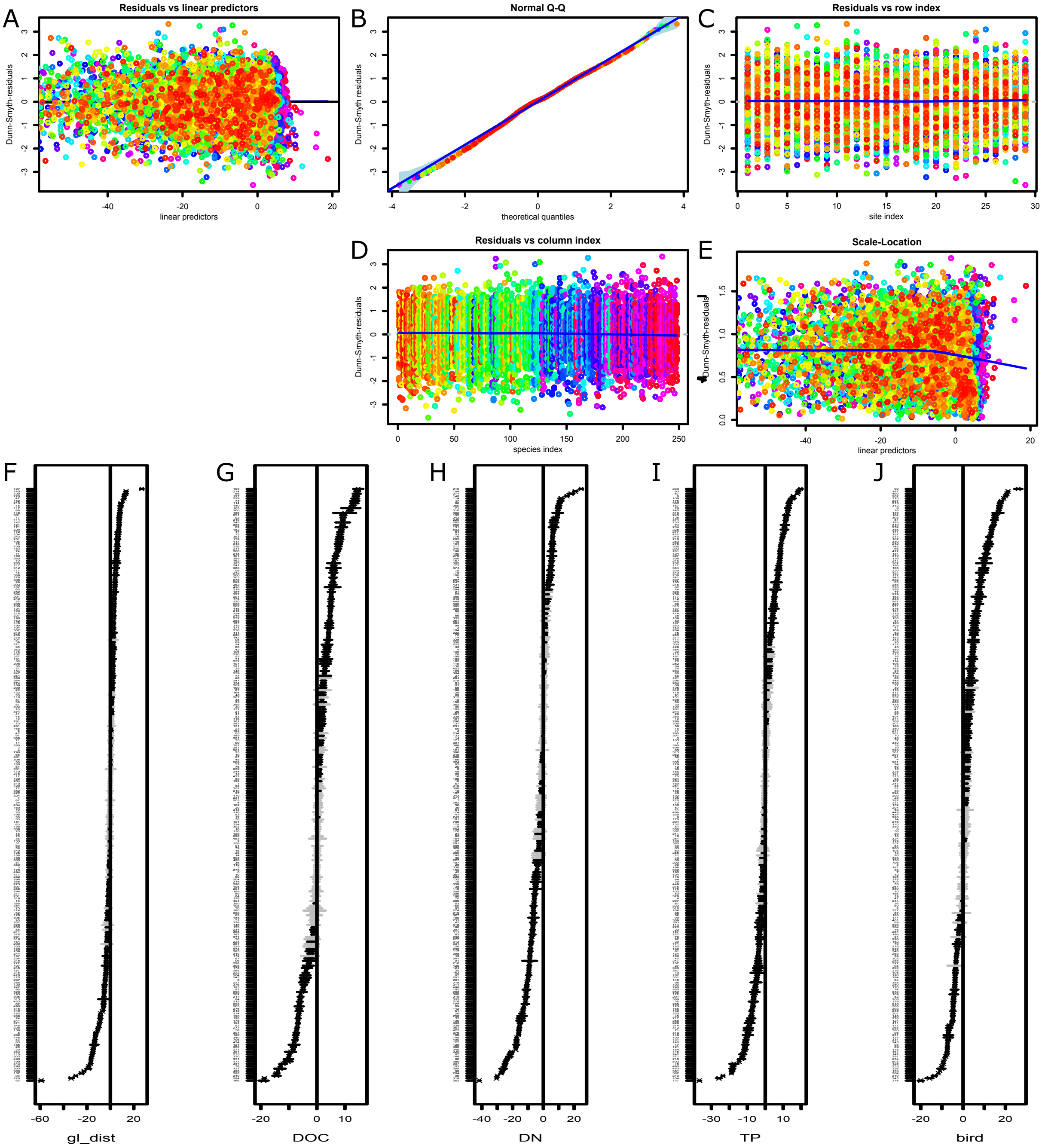

Supplementary Figure 7. Results from a general linear latent model (gllvm) on selected KEGG families.

Plots of Dunn-Smyth residuals, also known as randomized quantile residuals, are shown, including a plot of residuals against linear predictors of fitted values (A), a Normal Q-Q plot of residuals with a simulated point-wise 95% confidence interval envelope (B), residuals against row index (C) and column index (D) and scale-location plot (E). The estimated coefficients for predictors and their 95% confidence intervals (F – distance from glaciers (gl_dist), G – Dissolved organic carbon (DOC), H – Dissolved nitrogen (DN), I – Total phosphor (TP) and J – bird impact), allow to study the nature of effects of environmental variables on KEGG dynamics. Point estimates (ticks) for coefficients of the environmental variables and their 95% confidence intervals (lines) are given, with those colored in black denoting intervals not containing zero.

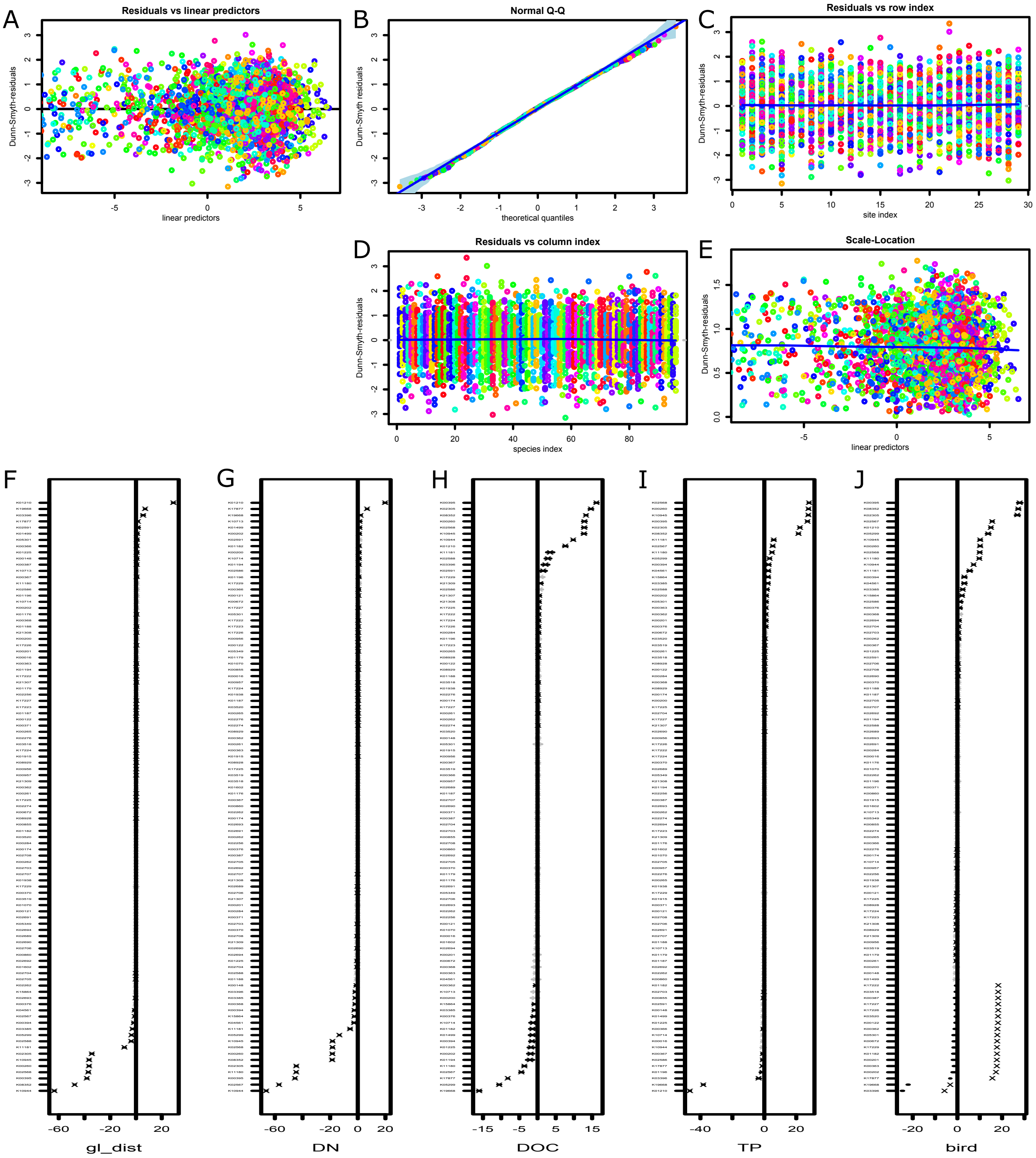

Supplementary Figure 8. Results from a general linear latent model (gllvm) on selected traits.
Plots of Dunn-Smyth residuals, also known as randomized quantile residuals, are shown, including a plot of residuals against linear predictors of fitted values (A), a Normal Q-Q plot of residuals with a simulated point-wise 95% confidence interval envelope (B), residuals against row index (C) and column index (D) and scale-location plot (E). The estimated coefficients for predictors and their 95% confidence intervals (F – distance from glaciers (gl_dist), G – Dissolved organic carbon (DOC), H – Dissolved nitrogen (DN)), allow to study the nature of effects of environmental variables on trait dynamics (see figure 3A-F for summary). Point estimates (ticks) for coefficients of the environmental variables and their 95% confidence intervals (lines) are given, with those colored in black denoting intervals not containing zero.

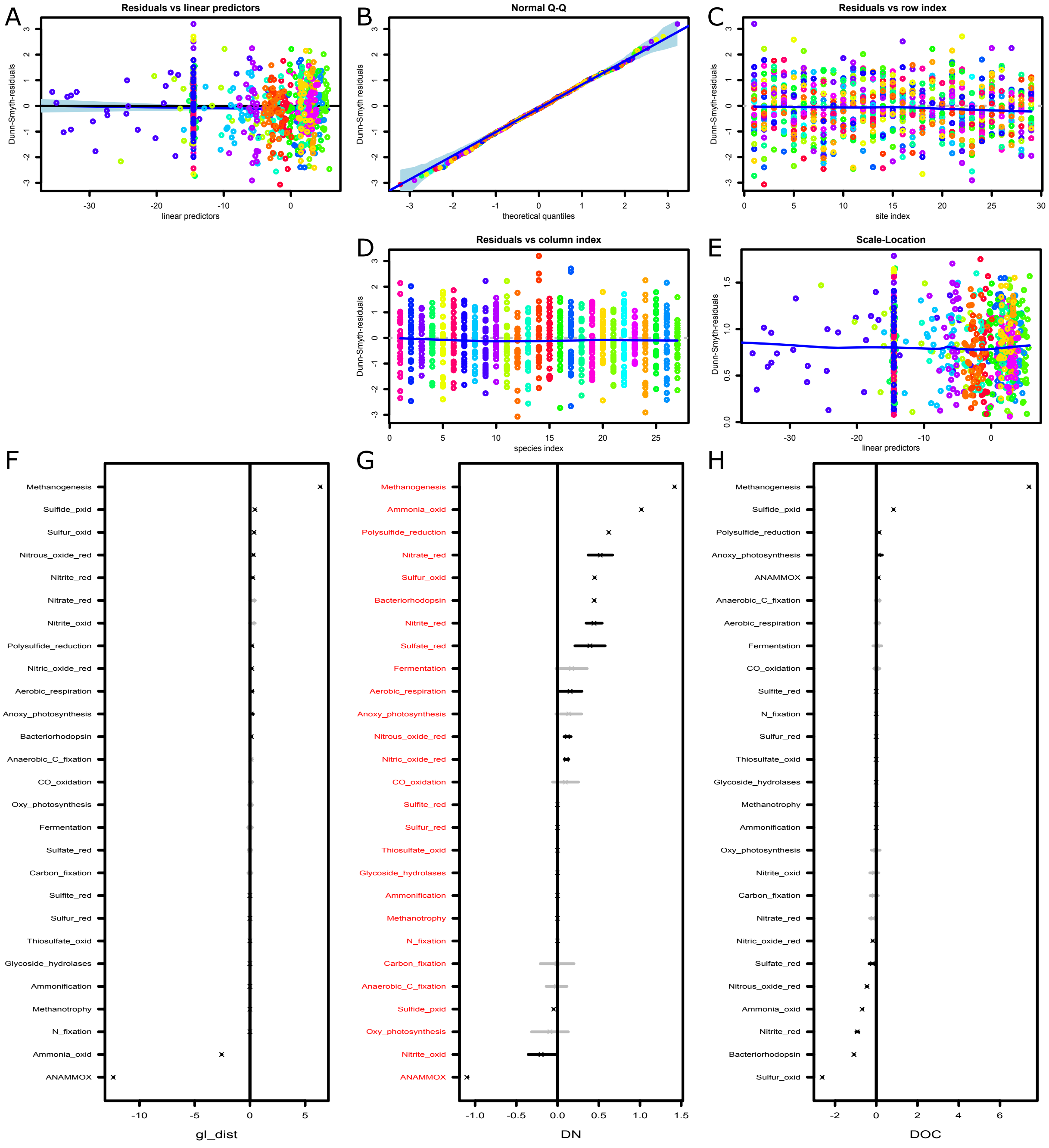

Supplementary Figure 9. Proportion of potential methanotrophic bacterial genera. Proportion of potential methanotrophic bacterial genera in the lake systems as determined by 16S rRNA gene sequencing.

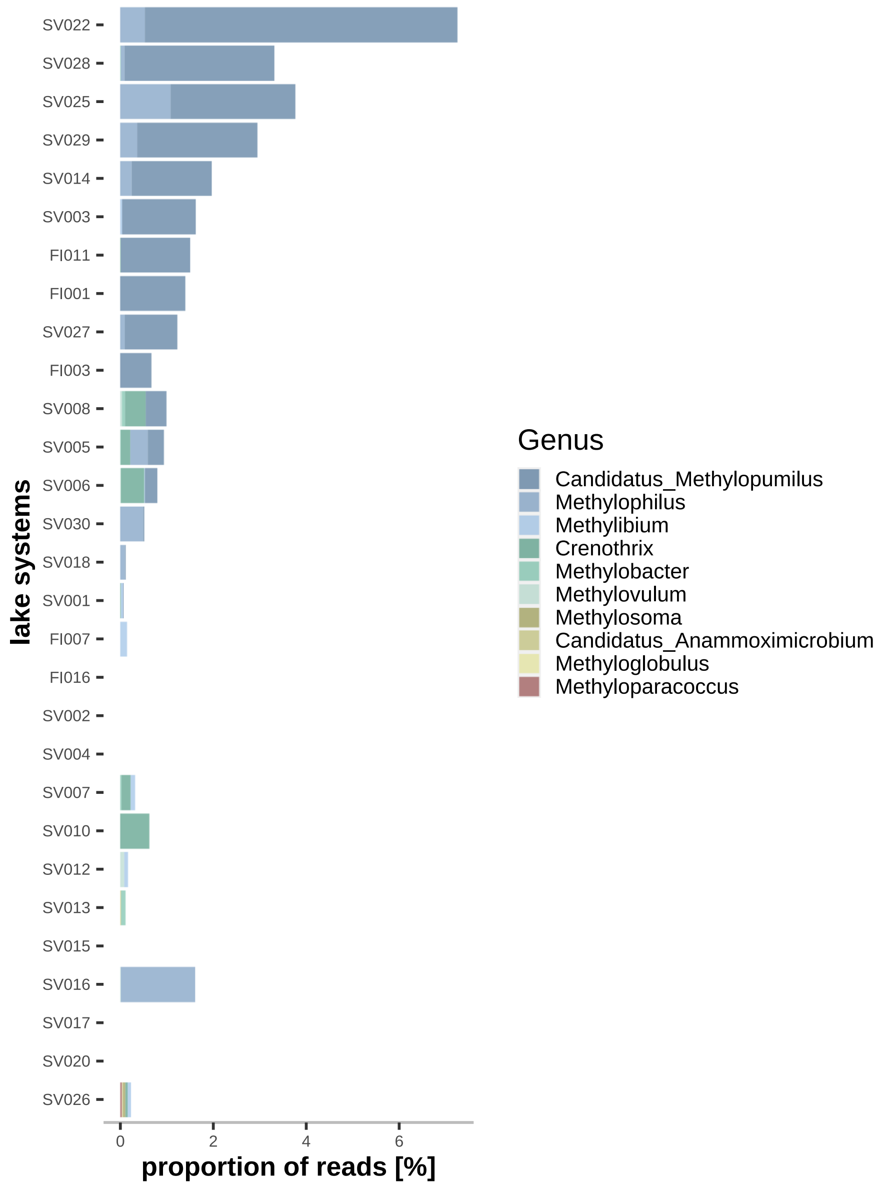

Supplementary Figure 10. Example for gating strategy.
First populations of cells were distinguished based on their forward (FSC) and side scatter (SSC) properties. Next, single parameter histograms and density plots were used on the fluorescence signals.

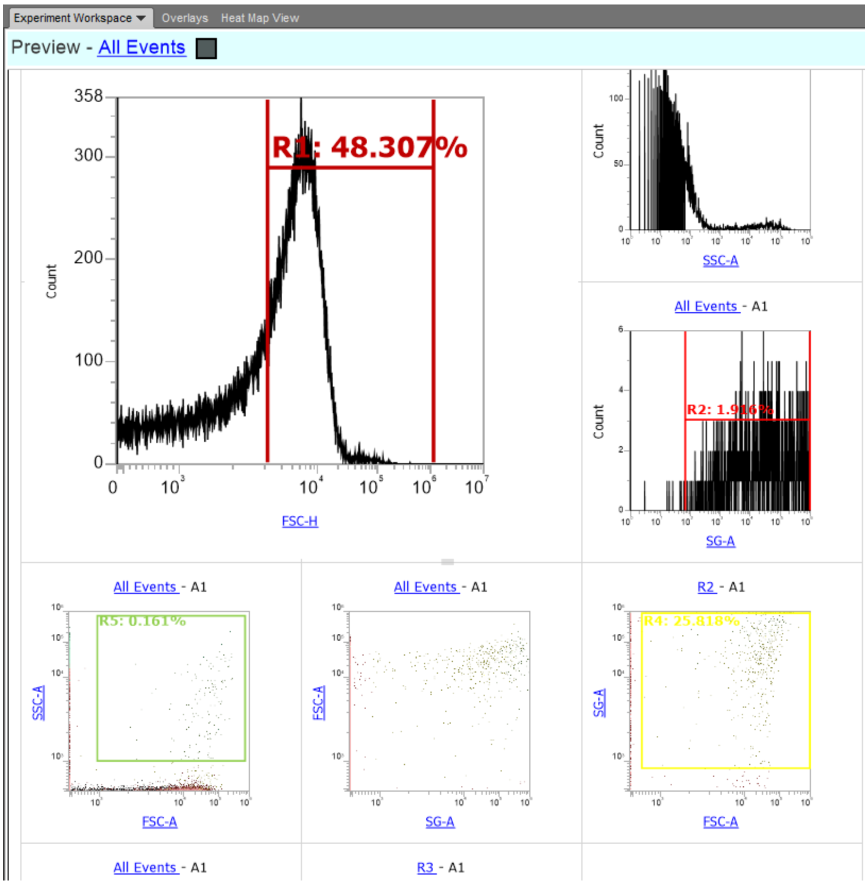

Supplement: Supplementary file 1 — Supplementary Information [file 41467_2023_38806_MOESM1_ESM.pdf]
