# Supplementary material for: Trajectories of freshwater microbial genomics and greenhouse gas saturation upon glacial retreat
Source: Nat Commun. 2023 Jun 3;14:3234. doi: 10.1038/s41467-023-38806-w (PMC10239486; doi:10.1038/s41467-023-38806-w)
Supplement: Supplementary file 3 — Reporting summary [file 41467_2023_38806_MOESM3_ESM.pdf]

nature portfolio

Corresponding author(s): Alexander Eiler

Last updated by author(s): Apr 18, 2023

Reporting Summary

Nature Portfolio wishes to improve the reproducibility of the work that we publish. This form provides structure for consistency and transparency in reporting. For further information on Nature Portfolio policies, see our Editorial Policies and the Editorial Policy Checklist.

Statistics

For all statistical analyses, confirm that the following items are present in the figure legend, table legend, main text, or Methods section.

n/a	Confirmed	
☐	☒	The exact sample size (*n*) for each experimental group/condition, given as a discrete number and unit of measurement
☐	☒	A statement on whether measurements were taken from distinct samples or whether the same sample was measured repeatedly
☐	☒	The statistical test(s) used AND whether they are one- or two-sided<br>*Only common tests should be described solely by name; describe more complex techniques in the Methods section.*
☐	☒	A description of all covariates tested
☐	☒	A description of any assumptions or corrections, such as tests of normality and adjustment for multiple comparisons
☐	☒	A full description of the statistical parameters including central tendency (e.g. means) or other basic estimates (e.g. regression coefficient) AND variation (e.g. standard deviation) or associated estimates of uncertainty (e.g. confidence intervals)
☐	☒	For null hypothesis testing, the test statistic (e.g. *F*, *t*, *r*) with confidence intervals, effect sizes, degrees of freedom and *P* value noted<br>*Give P values as exact values whenever suitable.*
☒	☐	For Bayesian analysis, information on the choice of priors and Markov chain Monte Carlo settings
☒	☐	For hierarchical and complex designs, identification of the appropriate level for tests and full reporting of outcomes
☐	☒	Estimates of effect sizes (e.g. Cohen's *d*, Pearson's *r*), indicating how they were calculated

Our web collection on statistics for biologists contains articles on many of the points above.

Software and code

Policy information about availability of computer code

Data collection	No software was used.
Data analysis	All code is provided on https://github.com/alper1976/chronosequences and metagenome analysis code is provided on https://github.com/jtamames/SqueezeMeta

For manuscripts utilizing custom algorithms or software that are central to the research but not yet described in published literature, software must be made available to editors and reviewers. We strongly encourage code deposition in a community repository (e.g. GitHub). See the Nature Portfolio guidelines for submitting code & software for further information.

Data

Policy information about availability of data

All manuscripts must include a data availability statement. This statement should provide the following information, where applicable:

- Accession codes, unique identifiers, or web links for publicly available datasets
- A description of any restrictions on data availability
- For clinical datasets or third party data, please ensure that the statement adheres to our policy

The raw demultiplexed sequence data (amplicon and metagenomic reads) have been uploaded to the Sequence Read Archive (SRA) with BioProject accession number PRJNA729725. The ASV tables, metadata (Supplementary table S and method descriptions have been deposited with the OSF data repository (www.osf.io) under DOI 10.17605/OSF.IO/PNWKS.

Research involving human participants, their data, or biological material

Policy information about studies with human participants or human data. See also policy information about sex, gender (identity/presentation), and sexual orientation and race, ethnicity and racism.

Reporting on sex and gender	*Use the terms sex (biological attribute) and gender (shaped by social and cultural circumstances) carefully in order to avoid confusing both terms. Indicate if findings apply to only one sex or gender; describe whether sex and gender were considered in study design; whether sex and/or gender was determined based on self-reporting or assigned and methods used. Provide in the source data disaggregated sex and gender data, where this information has been collected, and if consent has been obtained for sharing of individual-level data; provide overall numbers in this Reporting Summary. Please state if this information has not been collected. Report sex- and gender-based analyses where performed, justify reasons for lack of sex- and gender-based analysis.*
Reporting on race, ethnicity, or other socially relevant groupings	*Please specify the socially constructed or socially relevant categorization variable(s) used in your manuscript and explain why they were used. Please note that such variables should not be used as proxies for other socially constructed/relevant variables (for example, race/ethnicity should not be used as a proxy for socioeconomic status). Provide clear definitions of the relevant terms used, how they were provided (by the participants/respondents, the researchers, or third parties), and the method(s) used to classify people into the different categories (e.g. self-report, census or administrative data, social media data, etc.) Please provide details about how you controlled for confounding variables in your analyses.*
Population characteristics	*Describe the covariate-relevant population characteristics of the human research participants (e.g. age, genotypic information, past and current diagnosis and treatment categories). If you filled out the behavioural & social sciences study design questions and have nothing to add here, write "See above."*
Recruitment	*Describe how participants were recruited. Outline any potential self-selection bias or other biases that may be present and how these are likely to impact results.*
Ethics oversight	*Identify the organization(s) that approved the study protocol.*

Note that full information on the approval of the study protocol must also be provided in the manuscript.

Field-specific reporting

Please select the one below that is the best fit for your research. If you are not sure, read the appropriate sections before making your selection.

☐ Life sciences ☐ Behavioural & social sciences ☒ Ecological, evolutionary & environmental sciences

For a reference copy of the document with all sections, see nature.com/documents/nr-reporting-summary-flat.pdf

Ecological, evolutionary & environmental sciences study design

All studies must disclose on these points even when the disclosure is negative.

Study description	This study uses genomic and chemical data from 5 freshwater chronosequences in Arctic Svalbard and Alpine Norway to study the functional and taxonomic succession of the microbiome upon glacial retreat. Each chronosequence contained 4-6 lakes.
Research sample	Water samples from 31 freshwater lakes were taken in this study for DNA, nutrients, bacterial numbers, and chromophoric characteristics.
Sampling strategy	Five glacial chronosequences at Svalbard, Kongsfjorden (79º N) and Longyearbyen (78º N), and mainland Norway (Finse, 60º N) were studied. 31 lakes with minimal anthropogenic influence, spanning a distance of 0.2 to 10 km from the glaciers were chosen in these areas. During sampling, water samples were collected from at least three locations with a 4-meter-long grabber that was attached to a 1L bottle and pooled prior to subsampling for further measurements which require approximately 1.5 liters of water sample from each sampling site.
Data collection	Sampling-related data was collected in situ by the sampling team. Data generated after sampling were collected by technicians who are in charge of the corresponding measurements.
Timing and spatial scale	Water samples were taken from 31 lakes with a distance of 0.2 to 10 km from the glaciers, during summer 2019 (8th to 11th of August at Svalbard and 23rd of August at Finse). A map of the sampling locations is provided in the supplementary information.
Data exclusions	Samples SV011 and SV021 with insufficient reads to cover ASV richness were removed from rarefying step in the ecoinformatic analysis. Samples FI016 and SV011 did not give any libraries with acceptable quality, and thus these samples were excluded from metagenome sequencing.
Reproducibility	Samples were treated following the standard procedures that are stated in detail in the methods section. Gas samples from both in situ lake water and incubated water were taken in triplicates to verify the reproducibility of gas extraction methods.

Randomization | Sampling sites were chosen based on essentially the same biotic and abiotic history and were treated identically in this study. Thus, allocation is not relevant in this study.

Blinding | Blinding was not relevant since only environmental samples were taken in this study.

Did the study involve field work? ☒ Yes ☐ No

Field work, collection and transport

Field conditions | Details on field conditions during sampling campaign are provided in OSF data repository (www.osf.io) under DOI 10.17605/OSF.IO/PNWKS, and include temperature.

Location | Details on sample locations are given in OSF data repository (www.osf.io) under DOI 10.17605/OSF.IO/PNWKS.

Access & import/export | Access for field sampling at Svalbard was granted by Sysselmannan under document 19/01240-11.

Disturbance | Disturbance were minimized by accessing sampling sites per foot and using a 4 meter long grabber from the shoreline.

Reporting for specific materials, systems and methods

We require information from authors about some types of materials, experimental systems and methods used in many studies. Here, indicate whether each material, system or method listed is relevant to your study. If you are not sure if a list item applies to your research, read the appropriate section before selecting a response.

Materials & experimental systems

n/a	Involved in the study
☒	☐ Antibodies
☒	☐ Eukaryotic cell lines
☒	☐ Palaeontology and archaeology
☒	☐ Animals and other organisms
☒	☐ Clinical data
☒	☐ Dual use research of concern
☒	☐ Plants

Methods

n/a	Involved in the study
☒	☐ ChIP-seq
☐	☒ Flow cytometry
☒	☐ MRI-based neuroimaging

Flow Cytometry

Plots

Confirm that:

☒ The axis labels state the marker and fluorochrome used (e.g. CD4-FITC).

☒ The axis scales are clearly visible. Include numbers along axes only for bottom left plot of group (a 'group' is an analysis of identical markers).

☒ All plots are contour plots with outliers or pseudocolor plots.

☒ A numerical value for number of cells or percentage (with statistics) is provided.

Methodology

Sample preparation | Samples for bacterial counts were fixed with 37% borax buffered formaldehyde (final con- centration 2%) and stored at 4 °C prior to analyses. Cells were stained with the fluorescent nucleic acid stain SYBR green I (Molecular probes, Invitrogen, Waltham, Massachusetts, USA) for at least 30 minutes (1x final concentration).

Instrument | Attune® NxT Acoustic Focusing Cytometer (Thermo Fisher) equipped with an Invitrogen Attune NxT Autosampler equipped with a 488 nm laser using green fluorescence for triggered particle scoring.

Software | Attune Nxt Software

Cell population abundance | Details are reported in OSF data repository (www.osf.io) under DOI 10.17605/OSF.IO/PNWKS.

Gating strategy	Cultures of bacteria and algae were used to obtain the gating in FSC, SSC and fluorescence signals. We also used backgatng to confirm our gating strategy.

☒ Tick this box to confirm that a figure exemplifying the gating strategy is provided in the Supplementary Information.
